# Discovery of Steninae from Ningxia, Northwest China (Coleoptera, Staphylinidae)

**DOI:** 10.3897/zookeys.272.4389

**Published:** 2013-02-22

**Authors:** Liang Tang, Li-Zhen Li

**Affiliations:** 1 Department of Biology, Shanghai Normal University, 100 Guilin Road, 1st Educational Building 323 Room, Shanghai, 200234 P. R. China

**Keywords:** Coleoptera, Staphylinidae, Steninae, China, Ningxia, identification key, new species

## Abstract

A study on the Steninae of Ningxia Autonomous Region is presented. Sixteen species are recognized, including new province records for 11 species and four new species: *Stenus biwenxuani* **sp. n.**, *Stenus liupanshanus* **sp. n.**, *Dianous yinziweii* **sp. n.**, *Dianous ningxiaensis* **sp. n.** Habitus photos of the new species, illustrations of diagnostic characters of all species and a key to species of the Steninae recorded from Ningxia are provided.

## Introduction

Steninae, comprising two genera *Stenus* Latreille, 1797 and *Dianous* Leach, 1819, is a speciose subfamily of Staphylinidae. So far, 296 *Stenus* species and 103 *Dianous* species have been recorded from China. As far as the Steninae are concerned, Ningxia Autonomous Region is one of the most poorly explored regions, with merely two species recorded (Puthz 2008b): *Stenus deceptiosus* Puthz, 2008 and *Stenus comma* Leconte, 1863. In the summer of 2008, a team surveyed the insect fauna of the Liupan Shan Natural Reserve in southern Ningxia and collected a large number of Steninae. In this paper, we report the results of the study on that material, which includes two new *Stenus* and two new *Dianous* species, and new province records for eleven *Stenus* species.

## Material and methods

The specimens examined in this paper were collected by sifting leaf litter in forests and killed with ethyl acetate. For examination of the male genitalia, the last three abdominal segments were detached from the body after softening in hot water. The aedeagi, together with other dissected pieces, were mounted in Euparal (Chroma Gesellschaft Schmidt, Koengen, Germany) on plastic slides. Photos of sexual characters were taken with a Canon G7 camera attached to an Olympus SZX 16 stereoscope; habitus photos were taken with a Canon macro photo lens MP-E 65 mm attached to a Canon EOS40D camera.

Only records published after 2000 are given in the list of synonyms of each species. Articles published prior to 2001 may be found in Herman 2001.

The type specimens treated in this study are deposited in the following public and private collections:

**cPut** private collection V. Puthz, Schlitz, Germany

**SHNU** Department of Biology, Shanghai Normal University, P. R. China

The measurements of proportions are abbreviated as follows:

**BL** body length, measured from the anterior margin of the clypeus to the posterior margin of abdominal tergite X

**FL** forebody length, measured from the anterior margin of the clypeus to the apical margin of the elytra (apicolateral angle)

**HW** width of head including eyes

**PW** width of pronotum

**EW** width of elytra

**PL** length of pronotum

**EL** length of elytra, measured from humeral angle

**SL** length of elytral suture

## Taxonomy

### Key to the species of Steninae of Ningxia

**Table:** 

1	Labium unmodified; eyes relatively small, head mostly with distinct temples (*Dianous*)	2
–	Labium specialized, ejectable; eyes large, occupying entire lateral margin of head (*Stenus*)	4
2	First segment of metatarsus longer than the following segments combined; elytra without orange spots. Aedeagus: Fig. 24	*Dianous inaequalis inaequalis*
–	First segment of metatarsus not longer than the following segments combined; elytra each with an orange spot	3
3	Larger species, BL: 6.7mm; body with strong metallic luster, elytral spots larger and longitudinal. Sexual characters: Figs 49–52	*Dianous ningxiaensis*
–	Smaller species, BL: 4.8–5.1mm; body with faint metallic luster, elytral spots smaller and transverse. Sexual characters: Figs 42–48	*Dianous yinziweii*
4	Metatarsomere IV bilobed	5
–	Metatarsomere IV simple	*7*
5	Larger species (BL: 5.5–6.7 mm), elytra with pair of orange spots. Sexual characters: Figs 9, 10	*Stenus coronatus*
–	Smaller species with BL less than 4.5 mm, elytra without spots	6
6	Brachypterous species with elytra distinctly shorter than wide. BL: 2.6–2.7mm. Sexual characters: Figs 32–41	*Stenus liupanshanus*
–	Fully winged species with elytra longer than wide. BL: 3.2–4.0 mm. Sexual characters: Figs 11, 12	*Stenus trigonuroides*
7	Smaller species (BL: 2.5–3.1 mm), abdomen without paratergites. Sexual characters: Figs 13, 14	*Stenus pilosiventris*
–	Larger species with BL at least 3 mm, abdomen with paratergites	8
8	First three visible abdominal tergites with distinct basal keels	9
–	Abdominal tergites without basal keels	12
9	First three visible abdominal tergites with four basal keels. Aedeagus: Fig. 16	*Stenus melanarius melanarius*
–	First three visible abdominal tergites with three basal keels	10
10	Smaller species with elytra distinctly shorter than wide. BL: 3.0–3.2mm. Aedeagus: Fig. 15	*Stenus puthzi*
–	Larger species (BL at least 4.4 mm) with elytra longer than wide	11
11	Smaller species with reddish legs. BL: 4.4–4.7mm. Aedeagus: Fig. 18	*Stenus secretus*
–	Larger species with black legs. BL: 5.4–6.0 mm. Aedeagus: Fig. 17	*Stenus juno*
12	Elytra without orange spots. BL: 5.4 mm. Sexual characters: Figs 26–30	*Stenus biwenxuani*
–	Elytra with pair of orange spots	13
13	Legs reddish. BL: 4.8–5.2 mm. Aedeagus: Fig. 19	*Stenus alienus*
–	Legs black	14
14	Smaller species, BL: 3.5–4.3 mm, elytral punctation extremely dense. Aedeagus: Fig. 20	*Stenus scabratus*
–	Larger species with BL at least 4.3 mm, elytral punctation less dense	15
15	Lateral portions of frons sparsely punctate, interstices at least as wide as diameter of punctures. BL: 4.4–5.6 mm. Aedeagus: Fig. 21	*Stenus deceptiosus*
–	Lateral portions of frons densely punctate, interstices smaller than diameter of punctures	16
16	Male paratergites with punctures arranged in two irregular rows. BL: 4.3–5.5 mm. Aedeagus: Figs 18, 20, 21 in Puthz 2008	*Stenus comma*
–	Male paratergites with punctures mostly arranged in one irregular row. BL: 4.4–5.5 mm. Aedeagus: Figs 22, 23	*Stenus falsator*

#### Stenus coronatus

Benick, 1928

http://species-id.net/wiki/Stenus_coronatus

Figs 9, 10

##### Material examined:

**China: Ningxia:** 2 ♂♂, 4 ♀♀, Jinyuan County, Fengtai Linchang, 2310 m, 21.VI.2008, Wen-Xuan Bi leg.

##### Distribution.

China (Ningxia, Yunnan, Shanxi, Henan, Hebei, Beijing, Jilin), Korea, Japan.

#### Stenus trigonuroides

Zheng, 1993

http://species-id.net/wiki/Stenus_trigonuroides

Figs 11, 12

Stenus trigonuroidesi Zheng, 1993: 229; Puthz 2008a: 173.

##### Material examined:

**China: Ningxia:** 2 ♂♂, 2 ♀♀, Jinyuan County, Fengtai Linchang, 2300 m, 27–28.VI.2008, Wen-Xuan Bi leg.

##### Distribution.

China (Ningxia, Sichuan, Liaoning).

#### Stenus pilosiventris

Bernhauer, 1915

http://species-id.net/wiki/Stenus_pilosiventris

Figs 13, 14

##### Material examined:

**China: Ningxia:** 2 ♂♂, 1 ♀, Jinyuan County, Sutai Linchang, 2300m,21.VI.2008, Zi-Wei Yin leg.

##### Distribution.

China (Ningxia, Shanxi, Shanghai, Beijing), Korea, Mogolia, Russia.

#### Stenus puthzi

Hromádka, 1977

http://species-id.net/wiki/Stenus_puthzi

Fig. 15

Stenus puthzii Hromádka, 1977: 7.
Stenus asprohumilisi Zhao & Zhou, 2006: 284; Puthz 2008a: 151.

##### Material examined:

**China: Ningxia:** 1 ♂, 1 ♀, Jinyuan County, Heshangpu Linchang, 27.VI.2008, Zi-Wei Yin leg.

##### Distribution.

China (Ningxia, Shanxi, Heilongjiang), Russia.

#### Stenus melanarius melanarius

Stephens, 1833

http://species-id.net/wiki/Stenus_melanarius_melanarius

Fig. 16

##### Material examined:

**China: Ningxia:** 1 ♂, 2 ♀♀, Longde County, Sutai Linchang, 2200 m, 22.VI.2008, Zi-Wei Yin leg.

##### Distribution.

Widely distributed in the Palaearctic region.

**Figures 1–4.**
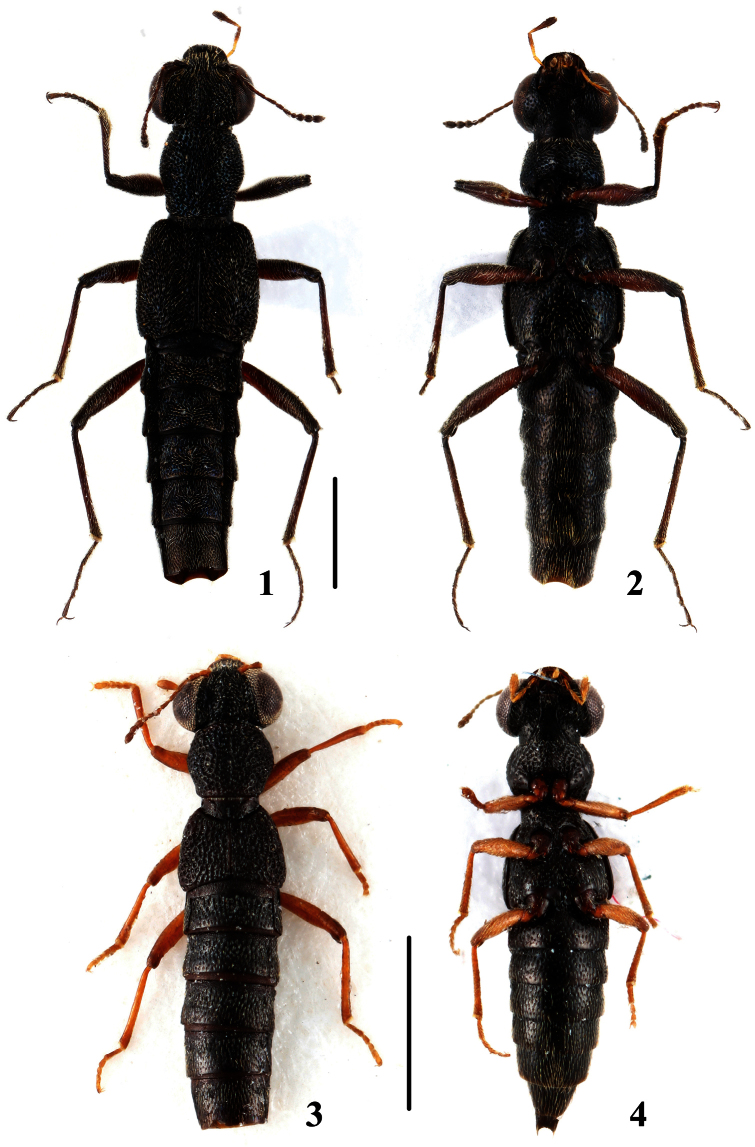
Habitus of *Stenus*. **1, 2** *Stenus biwenxuani* **3, 4** *Stenus liupanshanus*. Scales = 1 mm.

**Figures 5–8.**
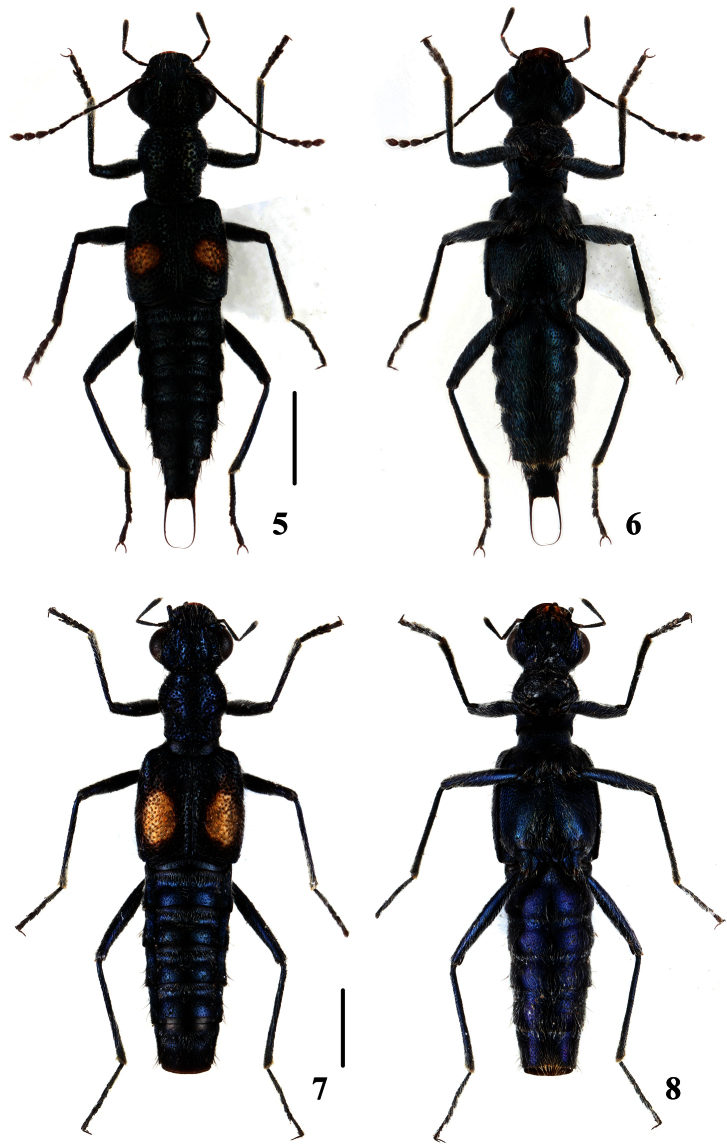
Habitus of *Dianous*. **5, 6** *Dianous yinziweii* **7, 8** *Dianous ningxiaensis*. Scales = 1 mm.

**Figures 9–16.**
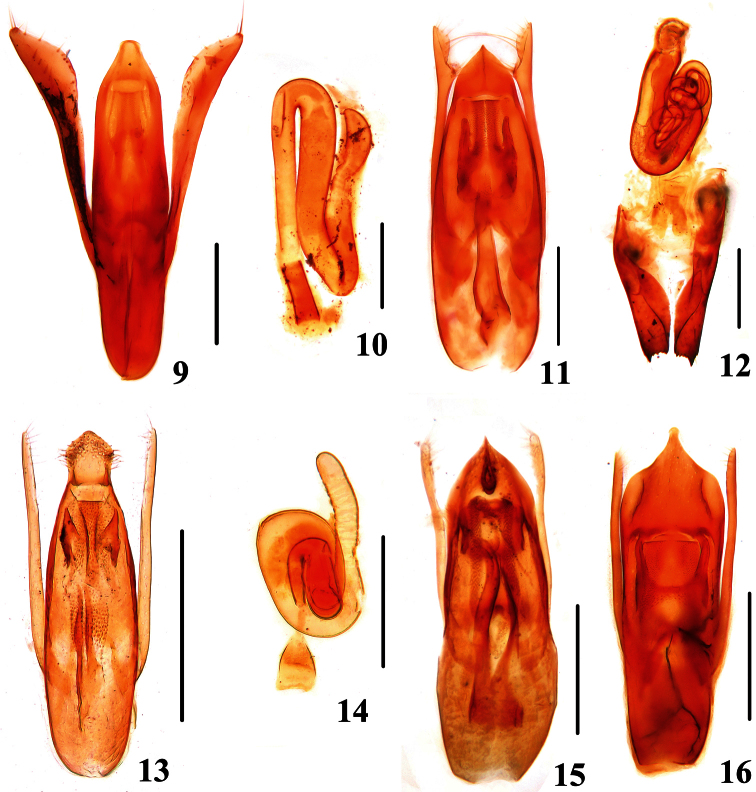
**9, 11, 13, 15, 16** Aedeagi of *Stenus*. **10, 12, 14** Spermathecae of *Stenus* **9, 10** *Stenus coronatus* **11, 12** *Stenus trigonuroides***13, 14** *Stenus pilosiventris* **15** *Stenus puthzi* **16** *Stenus melanarius melanarius*. Scales = 0.25 mm.

#### Stenus juno

Paykull, 1789

http://species-id.net/wiki/Stenus_juno

Fig. 17

##### Material examined:

**China: Ningxia:** 1 ♀, Jinyuan County, Fengtai Linchang, 2300 m, 27–28. VI.2008, Wen-Xuan Bi leg.

##### Distribution.

Widely distributed in the Holarctic region.

#### Stenus secretus

Bernhauer, 1915

http://species-id.net/wiki/Stenus_secretus

Fig. 18

##### Material examined:

**China: Ningxia:** 1 ♂, Jinyuan County, Guamagou Linchang, 2200 m, 4.VII.2008, Wen-Xuan Bi leg.

##### Distribution.

China (Ningxia, Heilongjiang, Shanxi), Korea, Russia.

#### Stenus alienus

Sharp, 1874

http://species-id.net/wiki/Stenus_alienus

Fig. 19

Stenus alienusi Sharp, 1874: 81; Puthz 2008b: 176.

##### Material examined:

**China: Ningxia:** 1 ♂, Jinyuan County, Qiuqianjia, 1800 m, 6-VI-2008, Wen-Xuan Bi leg.

##### Distribution.

China (Ningxia, Qinghai, Shaanxi, Shanxi, Beijing, Taiwan), Russia, Mogolia, Korea, Japan.

#### Stenus scabratus

Puthz, 2008

http://species-id.net/wiki/Stenus_scabratus

Fig. 20

Stenus scabratusi Puthz, 2008b: 180.

##### Material examined:

**China: Ningxia:** 3 ♂♂, 1 ♀, Jinyuan County, Hongxia Lingchang, 2000 m, 11–12.VI.2008, Wen-Xuan Bi leg.

##### Distribution.

China (Ningxia, Sichuan, Yunnan)

#### Stenus deceptiosus

Puthz, 2008

http://species-id.net/wiki/Stenus_deceptiosus

Fig. 21

Stenus deceptiosusi Puthz, 2008b: 184.

##### Material examined:

**China: Ningxia:** 7 ♂♂, 7 ♀♀, Jinyuan County, Qiuqianjia, 1800 m, 6.VII.2008, Wen-Xuan Bi leg.; 2 ♂♂, 3 ♀♀, Jingyuan County, Xixia, 15.VII. 2008, Feng Yuan leg.

##### Distribution.

China (Shaanxi, Shanxi, Ningxia, Hebei, Bejing, Liaoning), Korea.

#### Stenus falsator

Puthz, 2008

http://species-id.net/wiki/Stenus_falsator

Figs 22, 23

Stenus falsatori Puthz, 2008b: 182.

##### Material examined:

**China: Ningxia:** 8 ♂♂, 7 ♀♀, Jinyuan County, Qiuqianjia, 1800 m, 6.VII.2008, Wen-Xuan Bi leg.; 7 ♂♂, 8 ♀♀, Jingyuan County, Xixia, 15.VII. 2008, Feng Yuan leg.

##### Distribution.

China (Shanxi, Beijing, Jilin, Heilongjiang, Neimengu), Russia.

**Figure:**
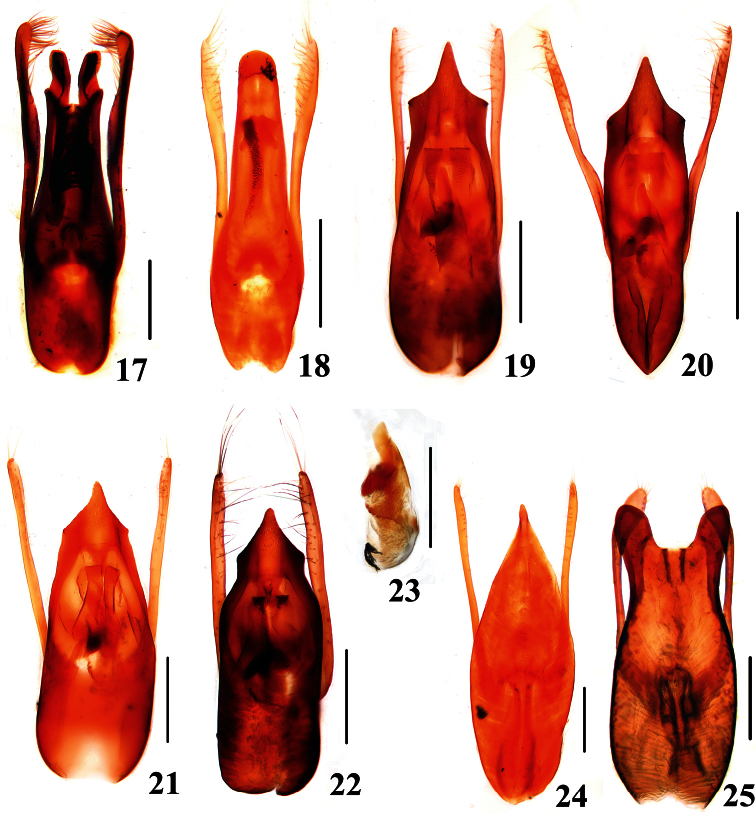
**Figures 17–25.** 17–22, 24, 25. Aedeagi of *Stenus* and *Dianous* **23** Internal plate of aedeagus of *Stenus*. **17** *Stenus juno* **18** *Stenus secretus* **19** *Stenus alienus***20** *Stenus scabratus* **21** *Stenus deceptiosus* **22, 23** *Stenus falsator***24** *Dianous inaequalis inaequalis* **25***Dianous chinensis*. Scales = 0.25 mm.

#### Stenus biwenxuani sp. n.

urn:lsid:zoobank.org:act:26EF1235-E3F4-427D-A3F8-31DFF08BFB05

http://species-id.net/wiki/Stenus_biwenxuani

Figs 1, 2 26–30

##### Type material.

**Holotype. China: Ningxia:** ♂, glued on a card with labels as follows: “Jinyuan County, Erlonghe Linchang, 2100 m, 9.VII.2008, Wen-Xuan Bi leg.” “Holotype / *Stenus biwenxuani*/ Tang & Li” [red handwritten label] (SHNU).

##### Diagnosis.

The new species belongs to the *Stenus comma* group, and is similar to *Stenus atrovestis* Puthz, 2008 (Puthz 2008b). However it can be easily distinguished from the latter by the reddish brown legs, longer elytra and simple metatibiae (*Stenus atrovestis* with black legs, shorter elytra and flattened metatibiae).

##### Description.

Body blackish with a faint plumbeous luster, antennae dark brown with club darker, maxillary palpi yellowish with last and apical half of penultimate segments brownish, legs reddish brown except knee darker with a faint plumbeous luster.

BL: 5.4 mm; FL: 2.7 mm.

HW: 1.00 mm, PL: 0.85 mm, PW: 0.80 mm, EL: 1.15 mm, EW: 1.11 mm, SL: 0.93 mm.

Head 0.90 times as wide as elytra; interocular area with deep longitudinal furrows, median portion moderately convex, not reaching the level of inner eye margins; punctures round, extremely dense, and of similar size; diameter of punctures about as wide as apical cross section of antennal segment III; interstices much narrower than half the diameter of punctures except those along the midline of the convex median portion, which may be a little broader than half the diameter of punctures. Antennae, when reflexed, extending a little beyond middle of pronotum; relative length of antennal segments from base to apex as 12 : 10 : 17.5 : 10: 9 : 6.5 : 7: 5 : 6 : 6 : 10. Paraglossa oval.

Pronotum 1.06 times as long as wide; disc with shallow and broad median longitudinal furrow fused with pairs of shallow impressions in anterior half, in the middle, and in posterior half; punctures round and very dense, slightly confluent, a little larger than those of head; interstices partially reticulated, of variable width, as wide as half the diameter of punctures or narrower.

Elytra 1.04 times as long as wide; disc slightly uneven with indistinct longitudinal humeral impression, indistinct postero-lateral impression, and indistinct sutural impression; punctures mostly confluent, a little larger than those of pronotum with rugose interstices.

Hind tarsi 0.76 times as long as hind tibiae, tarsomeres IV simple.

Abdomen semi-cylindrical with broad, raised and densely punctate paratergites of segments III–VI, width of paratergites of segment III slightly broader than apical width of metatibiae, punctures slightly larger than those on median portion of tergites; posterior margin of tergite VII with palisade fringe; punctures on abdominal tergites III–VIII round to elliptic, very dense, gradually becoming smaller posteriad; interstices mostly as wide as half the diameter of punctures at most, with relatively faint reticulation on all abdominal tergites.

**Figures 26–31.**
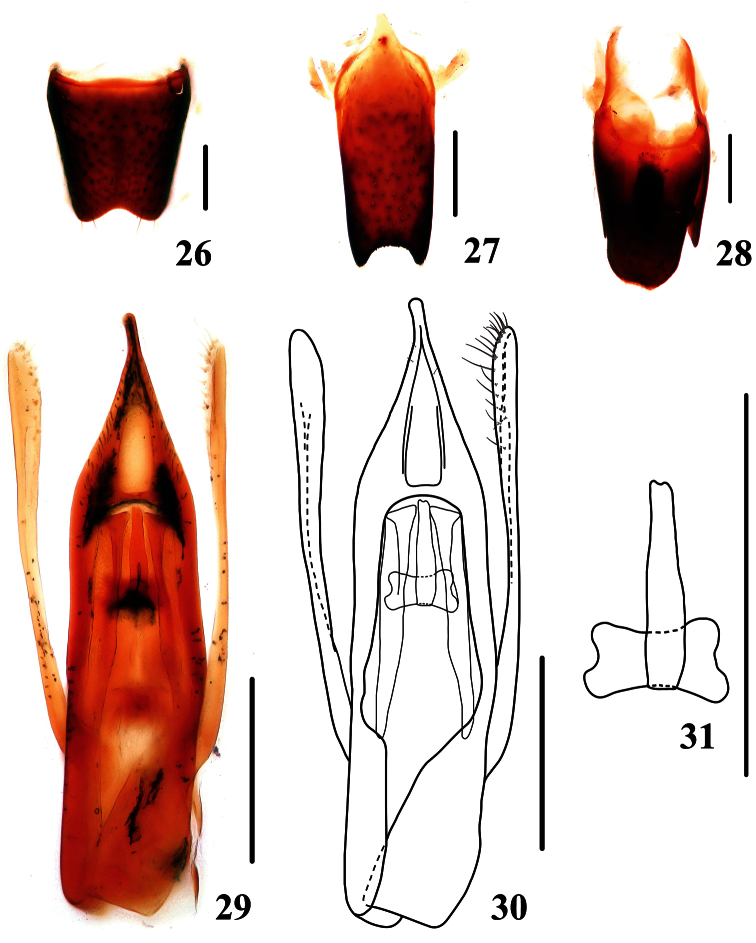
*Stenus biwenxuani*. **26** male sternite VIII **27** male sternite IX **28** male tergites IX, X **29, 30** aedeagus **31** sclerotized plate of aedeagus. Scales = 0.25 mm.

Male. Mesotibiae and metatibiae each with a subapical tooth on inner side; sternite VI impressed postero-medially with a shallow emargination along the posterior margin of the impression; sternite VII impressed medially, posterior margin of this impression emarginate; sternite VIII (Fig. 26) with emargination at middle of posterior margin; sternite IX (Fig. 27) with apico-lateral projections long and stout, posterior margin serrate; tergite X (Fig. 28) with posterior margin slightly emarginated. Aedeagus (Figs 29, 30) slender, median lobe with a very long and pointed apex; internal plate strongly sclerotized (Fig. 31), parameres extending beneath apex of median lobe, widened and folded in apical third, each with 18 setae on inner side.

Female. unknown.

##### Distribution.

China (Ningxia).

##### Etymology.

This species is named in honor of Mr. Wen-Xuan Bi, the collector of the new species.

#### Stenus liupanshanus sp. n.

urn:lsid:zoobank.org:act:3AA00430-6919-4876-B0DD-AAC1926A26C3

http://species-id.net/wiki/Stenus_liupanshanus

Figs 3, 4 32–41

##### Type material.

**Holotype.** **China: Ningxia:** ♂, glued on a card with labels as follows: “Jinyuan County, Fengtai Linchang, 2400 m, 26.VI.2008, Wen-Xuan Bi & Zi-Wei Yin leg.”“Holotype / *Stenus liupanshanus* / Tang & Li” [red handwritten label] (SHNU). **Paratypes.** 1 ♀, same data as for the holotype (SHNU); 1 ♂: ibidem, 2310m, 22.VI.2008, idem (cPut); 2 ♂♂, Jinyuan County, Dongshanpo, 2310 m, 27.VI.2008, Wen-Xuan Bi leg. (SHNU); 2 ♀♀, Jinyuan County, Heshangpu Linchang, 2300 m, 27.VI.2008, Wen-Xuan Bi leg. (SHNU); 2 ♀♀, Jinyuan County, Qiuqianjia, 1800 m, 6.VII.2008, Wen-Xuan Bi leg. (SHNU)

##### Diagnosis.

The new species belongs to the *Stenus cephalotes* group and can easily be distinguished from other Chinese representatives of this group by the presence of distinct reticulation on the forebody and the very short elytra (EL/EW less than 0.8).

##### Description.

Brachypterous; body dark brown with head slightly darker, anterior margin of labrum, antennae, maxillary palpi and legs reddish brown.

BL: 2.6–2.7mm; FL: 1.3 mm.

HW: 0.62–0.67 mm, PL: 0.44–0.48 mm, PW: 0.49–0.52 mm, EL: 0.45–0.51 mm, EW: 0.60–0.64 mm, SL: 0.32–0.34 mm.

Head 1.02–1.04 times as wide as elytra; interocular area with deep longitudinal furrows, median portion convex, slightly extending beyond the level of inner eye margins; punctures round, moderately confluent, and of similar size, diameter of punctures about as wide as apical cross section of antennal segment III; interstices rugose with indistinct reticulation, much narrower than half the diameter of punctures except those along the midline of the convex median portion, where they may be slightly broader than diameter of punctures. Antennae, when reflexed, not reaching middle of pronotum; relative length of antennal segments from base to apex as 6: 5.5: 8: 5: 5: 4: 3: 2.5: 4: 4: 5.5. Paraglossa oval.

Pronotum 0.91–0.93 times as long as wide; disk somewhat flattened, with shallow median longitudinal furrow; punctures slightly confluent, a little larger than those of head; interstices reticulated, distinctly narrower than half the diameter of punctures.

Elytra 0.75–0.79 times as long as wide, distinctly constricted at base; lateral margins gently divergent posteriad; disk rather even, suture slightly convex; punctation and interstices similar to those of pronotum.

Legs with hind tarsi 0.68 times as long as hind tibiae, tarsomeres IV distinctly bilobed.

Abdomen cylindrical; distinct paratergites absent, but rudimentary lateral border present; posterior margin of tergite VII with palisade fringe; punctures of abdominal tergites III–VIII elliptic, gradually becoming smaller posteriad; interstices narrower than half the diameter of punctures, with relatively faint microsculpture on tergites III–VII and distinct reticulation on tergites VIII–X.

**Figures 32–41.**
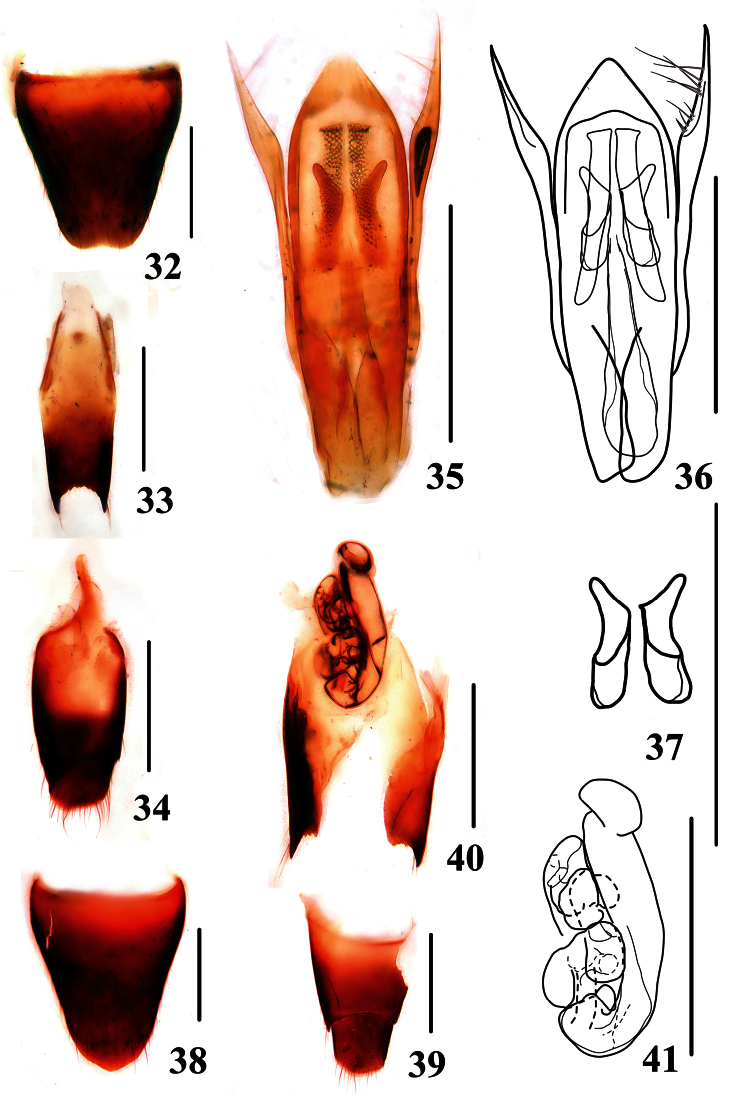
*Stenus liupanshanus*. **32** male sternite VIII **33** male sternite IX **34** male tergites IX, X **35, 26** aedeagus **37** expulsion hooks **38** female sternite VIII **39** female tergites IX, X **40** valvifers and spermatheca **41** spermatheca. Scales = 0.25 mm.

Male. Sternite VIII (Fig. 32) with very shallow emargination at middle of posterior margin; sternite IX (Fig. 33) with apicolateral projections very long and posterior margin serrate and emarginate; tergite X (Fig. 34) with posterior margin convex. Aedeagus (Figs 35, 36) with median lobe roundly pointed at apex; expulsion hooks (Fig. 37) very large; parameres extending a little beyond apex of median lobe, dilated in apical third, each with two groups of setae on inner side: 5–6 apical setae and 5–6 subapical setae.

Female. Abdomen broader than that of male; sternite VIII (Fig. 38) slightly produced in the middle of posterior margin; tergite X (Fig. 39) similar to that of male; sclerotized spermatheca bent twice with many bubble structures on second tube (Figs 40, 41).

##### Distribution.

China (Ningxia).

##### Etymology.

The specific name is derived from “Liupanshan”, the mountain where the type specimens were found.

#### Dianous inaequalis inaequalis

Champion, 1919

http://species-id.net/wiki/Dianous_inaequalis_inaequalis

Fig. 24

Dianous inaequalisi Champion, 1919: 45.
Dianouscaeruleoguttatusi Cameron, 1927: 6, 8.

##### Material examined:

**China: Ningxia:** 1 ♂, 2 ♀♀, Jinyuan County, Qiuqianjia, 1800 m, 6.VI.2008, Wen-Xuan Bi leg.

##### Distribution.

China (Yunnan, Sichuan, Ningxia), India.

#### Dianous yinziweii sp. n.

urn:lsid:zoobank.org:act:DC4E723C-CE73-4E38-88B1-168948840CD8

http://species-id.net/wiki/Dianous_yinziweii

Figs 5, 6 42–48

##### Type material.

**Holotype. China: Ningxia:** ♂, glued on a card with labels as follows: “Jinyuan County, Erlonghe Linchang, Xiaonanchuan, 2000 m, 10.VII.2008, Zi-Wei Yin leg.” “Holotype / *Dianous yinziweii* / Tang & Li” [red handwritten label] (SHNU). **Paratypes.** 155 ♂♂, 129 ♀♀, same data as for the holotype (2 pair in cPut, remainder in SHNU); 18 ♂♂, 28 ♀♀, Jingyuan County, Erlonghe Linchang, 2200 m, 22.VII. 2008, Feng Yuan leg. (SHNU); 1 ♂, Jinyuan County, Fengtai Linchang, 2400 m, 26.VI.2008, Wen-Xuan Bi leg. (SHNU)

##### Diagnosis.

The new species belongs to the *Dianous chinensis* complex and is similar to *Dianous banghaasi* Bernhauer, 1915 in sharing the elytral spots reaching the lateral margins in dorsal view. However, it can be easily distinguished from the latter by the distinctly smaller body size and the faint metallic luster of the entire body, which is strongly metallic blue in *Dianous banghaasi*.

##### Description.

Body black with a plumbeous luster, antennal club brownish, elytra each with a large transverse orange spot, which reaches the lateral margins of the elytra in dorsal view, and with a narrow band of coppery luster around the spot, pubescence silvery to golden brown throughout, that of elytral spots golden brown.

BL: 4.8–5.1mm; FL: 2.5–2.8 mm.

HW: 0.98–1.04 mm, PL: 0.83–0.85 mm, PW: 0.77–0.82 mm, EL: 1.17–1.22 mm, EW: 1.07–1.16 mm, SL: 0.98–1.00 mm

Head 0.85–0.94 times as wide as elytra; interocular area with deep longitudinal furrows, median portion convex; punctures round, slightly confluent along the furrows, larger and sparser in median area than those near inner margins of eyes, diameter of large punctures about as wide as apical cross section of antennal segment III; interstices smooth, much narrower than half the diameter of punctures. Antennae, when reflexed, extending distinctly beyond posterior margin of pronotum; relative length of antennal segments from base to apex as 13.5: 9: 20: 14: 13.5:12: 11.5: 11: 11: 10: 12.5.

Pronotum 1.03–1.08 times as long as wide; disk relatively even; punctures round, transversely confluent in posterior portion, a little larger than those on head; interstices smooth, narrower than half the diameter of punctures except those in median portion, which may be as broad as two or three punctures.

Elytra 1.03–1.09 times as long as wide; punctation and interstices similar to those of pronotum, except that punctation of basal half portion and along suture is distinctly confluent with rugose interstices.

Hind tarsi with tarsomeres IV distinctly bilobed.

Abdomen semi-cylindrical with broad, raised and densely punctate paratergites of segments III–VI, width of paratergites of segment III as broad as apical width of metatibiae, punctures minute; posterior margin of tergite VII with palisade fringe; punctures on abdominal tergites III–VIII minute, smaller than ommatidia of eyes; interstices without microreticulation except tergite VIII, varied from a little narrower than half the diameter of punctures to much broader than diameter of punctures.

Pubescence of fore body long and suberect, single setae as long as fourth antennal segment.

**Figures 42–48.**
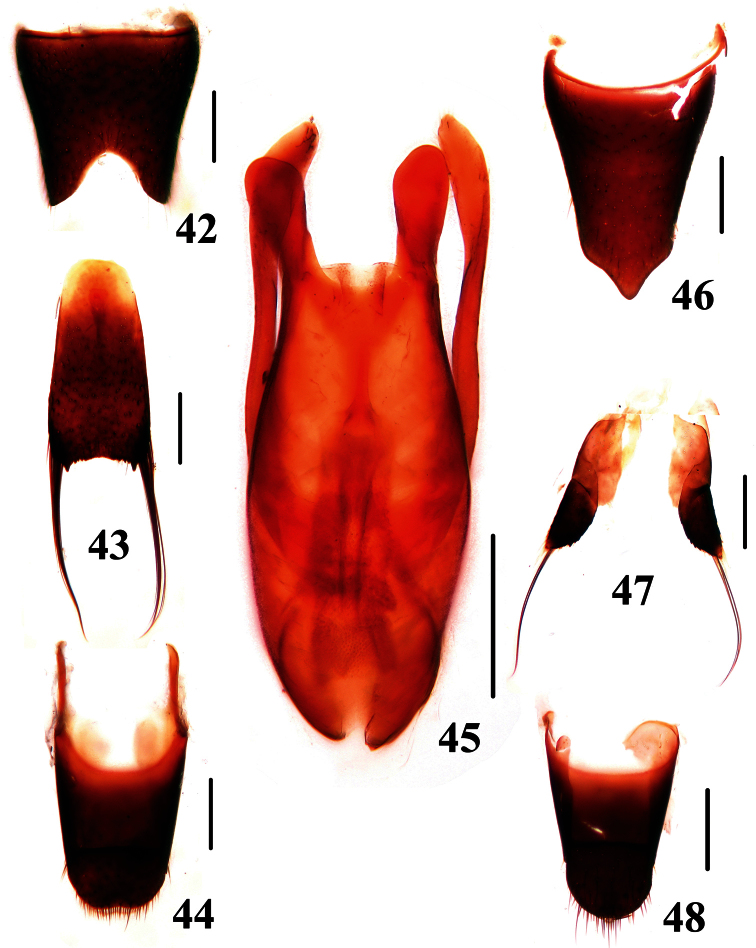
*Dianous yinziweii*. **42** male sternite VIII **43** male sternite IX **44** male tergites IX, X **45** aedeagus **46** female sternite VIII **47** valvifers **48** female tergites IX, X. Scales = 0.25 mm.

Male. Sternite VII impressed postero-medially with shallow emargination along posterior margin of the impression; sternite VIII (Fig. 42) with deep emargination in the middle of posterior margin; sternite IX (Fig. 43) with apicolateral projections moderately pointed and posterior margin serrate; tergite X (Fig. 44) with posterior margin slightly emarginated. Aedeagus (Fig. 45) with median lobe bilobed at apex; parameres slightly bent inwards, extending distinctly beyond the apex of median lobe, with setae on inner side of apical portion.

Female. Abdomen slightly broader than that of male; sternite VIII (Fig. 46) distinctly produced in the middle of posterior margin; valvifer (Fig. 47) with posterior margin finely serrate; tergite X (Fig. 48) with posterior margin convex.

##### Distribution.

China (Ningxia).

##### Etymology.

This species is named in honor of Mr. Zi-Wei Yin, the collector of the new species.

#### Dianous ningxiaensis sp. n.

urn:lsid:zoobank.org:act:25C5CED3-6704-4985-93FB-8E2FBB45C32E

http://species-id.net/wiki/Dianous_ningxiaensis

Figs 7, 8 49–52

##### Type material.

**Holotype. China: Ningxia:** ♂, glued on a card with labels as follows: “Jinyuan County, Erlonghe Linchang, Xiaonanchuan, 2000 m, 10.VII.2008, Zi-Wei Yin leg.” “Holotype / *Dianous ningxiaensis*/ Tang & Li” [red handwritten label] (SHNU).

##### Diagnosis.

The new species belongs to the *Dianous chinensis* complex and is similar to *Dianous chinensis* Bernhauer, 1915 (Fig. 25). It can be easily distinguished from the latter by the extremely large elytral spots and distinctly longer parameres of the aedeagus.

##### Description.

Body black with a blue to purple metallic luster, antennal club brownish, each elytron with a large elongate orange spot, which is 1/2 as long as and 3/5 as broad as the respective elytron, and with a coppery luster around the spot, pubescence silvery to golden brown throughout, that of elytral spots golden brown.

BL: 6.7 mm; FL: 3.3 mm.

HW: 1.07 mm, PL: 0.91 mm, PW: 0.87 mm, EL: 1.55 mm, EW: 1.40 mm, SL: 1.28 mm.

Head 0.77 times as wide as elytra; interocular area with deep longitudinal furrows, median portion convex; punctures round, similar in size, diameter of punctures about as wide as basal cross section of antennal segment III; interstices without microsculpture and of variable width, ranging from being narrower than half the diameter of punctures to being of similar width as diameter of punctures. Antennae, when reflexed, extending distinctly beyond posterior margin of pronotum; relative length of antennal segments from base to apex as 16: 9.5: 34.5: 16.5: 18: 15.5: 15.5: 15: 14: 12: 13.

Pronotum 1.04 times as long as wide; disk uneven, with two deep median impressions fused with a distinct basal impression; punctures mostly well delimited, slightly larger than those on head; interstices without microreticulation and of variable width.

Elytra 1.11 times as long as wide; punctation and interstices similar to those of pronotum except for a few larger punctures and partly fainly microsculptured interstices.

Hind tarsi with tarsomeres IV distinctly bilobed.

Abdomen semi-cylindrical with broad, raised and densely punctate paratergites of segments III–VI, paratergites of segment III slightly broader than apical width of metatibiae, punctures minute; posterior margin of tergite VII with palisade fringe; punctures on abdominal tergites III–VIII minute, smaller than ommatidia of eyes; interstices without microsculpture, except those of sternite VIII and of variable width.

Pubescence of fore body conspicuously long and suberect, single setae as long as fourth antennal segment.

**Figures 49–52.**
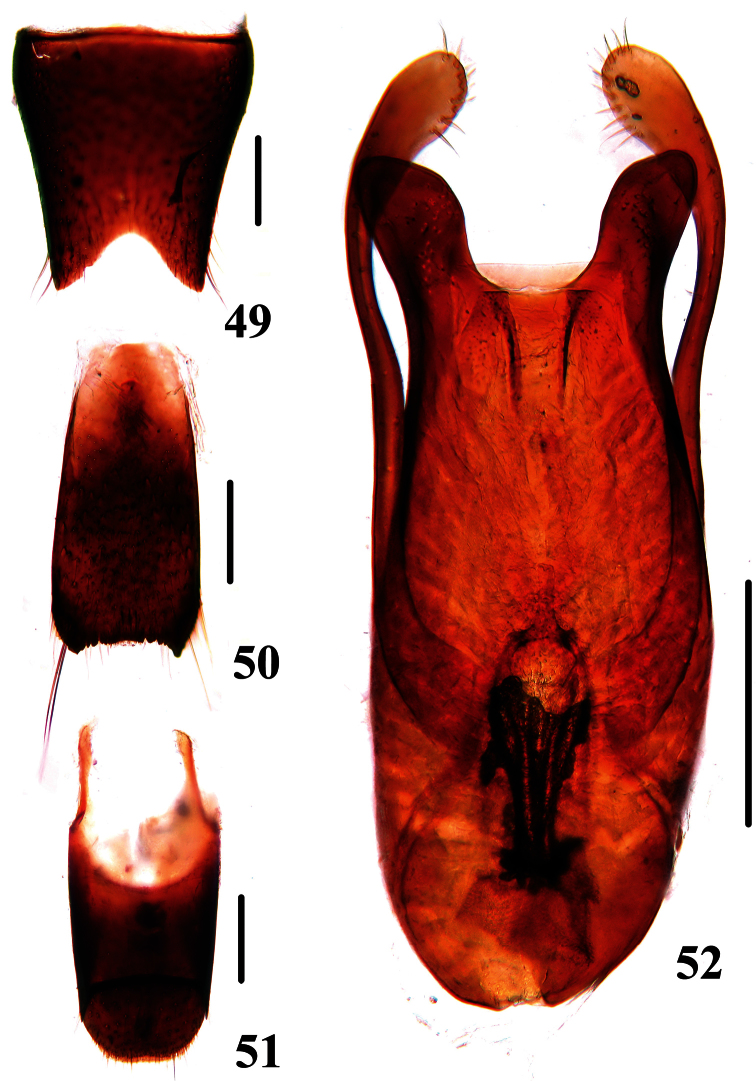
*Dianous ningxiaensis*. **49** male sternite VIII **50** male sternite IX **51** male tergites IX, X **52** aedeagus. Scales = 0.25 mm.

Male. Sternite VII with posteromedian portion slightly flattened and densely pubescent; sternite VIII (Fig. 49) with deep emargination in the middle of posterior margin; sternite IX (Fig. 50) with apicolateral projections moderately pointed and posterior margin serrate; tergite X (Fig. 51) with posterior margin slightly emarginated. Aedeagus (Fig. 52) with median lobe bilobed at apex; parameres bent inwards, extending distinctly beyond apex of median lobe, with setae on inner side of apical portion.

Female. Unknown.

##### Distribution.

China (Ningxia).

##### Etymology.

The specific name is derived from “Ningxia”, the type locality of this species.

## Supplementary Material

XML Treatment for Stenus coronatus

XML Treatment for Stenus trigonuroides

XML Treatment for Stenus pilosiventris

XML Treatment for Stenus puthzi

XML Treatment for Stenus melanarius melanarius

XML Treatment for Stenus juno

XML Treatment for Stenus secretus

XML Treatment for Stenus alienus

XML Treatment for Stenus scabratus

XML Treatment for Stenus deceptiosus

XML Treatment for Stenus falsator

XML Treatment for Stenus biwenxuani

XML Treatment for Stenus liupanshanus

XML Treatment for Dianous inaequalis inaequalis

XML Treatment for Dianous yinziweii

XML Treatment for Dianous ningxiaensis
